# Antibiotic Susceptibility Patterns and Prevalence of Some Extended Spectrum Beta-Lactamases Genes in Gram-Negative Bacteria Isolated from Patients Infected with Urinary Tract Infections in Al-Najaf City, Iraq

**Published:** 2019

**Authors:** Heba Takleef Majeed, Ahmed Abduljabbar Jaloob Aljanaby

**Affiliations:** Department of Microbiology, Faculty of Science, University of Kufa, Kufa, Iraq

**Keywords:** Iraq, Chronic kidney disease, *SHV*, *TEM*, Urinary tract infections

## Abstract

**Background:**

Urinary Tract Infection (UTI) in patients with Chronic Kidney Disease (CKD) caused by multi-drug resistance and Extended Spectrum Beta Lactamase (ESBL)-producing gram-negative bacteria has been increased in different countries. The aim of the present study was to detect the antibiotic susceptibility patterns and the distribution of *Bla-TEM*, *Bla-SHV* and *Bla-CTX-M* genes in gram-negative bacteria isolated from outpatients infected with UTI, with and without CKD in Al-Najaf city, Iraq.

**Methods:**

A total of 120 non-duplicate urine samples were collected from outpatients (37 male and 83 female) infected with UTI in Al-Najaf city, Iraq; 60 samples from patients Without Kidney Disease (WKD) and 60 samples from patients with CKD. The antibiotic susceptibility testing was done according to Kirby-Bauer method. PCR technique was performed to investigate the prevalence of *Bla-TEM*, *Bla-SHV* and *Bla-CTX-M* genes.

**Results:**

A total of 126 different gram-negative bacterial strains were isolated. *Escherichia coli (E. coli)* was the most prevalent bacterium (49 isolates) followed by *Idebsiella pneumonia (K. pneumonia*) (35 isolates), *Pseudomonas aeruginosa (P. aeruginosa)* (18 isolates), *Citrobacter freundii (C. freundii)* (12 isolates), *Enterobocter aerogenes (E. aerogenes)* (8 isolates) and *Proteus mirabilis (P. mirabilis)* (4 isolates). All bacterial isolates from UTI patients with CKD were resistant to antibiotics and carried *Bla-TEM*, *Bla-SHV* and *Bla-CTX-M* genes more than isolates from UTI patients with WKD.

**Conclusion:**

This study demonstrated that all bacterial isolates from UTI patients with CKD were more virulent than isolates from UTI patients with WKD.

## Introduction

Gram-negative bacteria are a common cause of Urinary Tract Infection (UTI), especially in older individuals and in patients infected with Chronic Kidney Disease (CKD). Urinary tract infection is one of the most important recurrent diseases worldwide especially in Middle East countries and the second most common illness in both males and females with prevalence of 1/2, respectively 1,2. Chronic kidney disease is a clinical syndrome characterized by defect in kidney glomerular filtration resulting in decreased clearance of metabolic waste product of the blood 3. About 6 to 40% of patients with CKD are susceptible to infection with UTI caused by Extended-Spectrum β-Lactamase (ESBL)-producing gram-negative bacteria worldwide 4. Annually, about more than 150 million cases are infected with UTI worldwide 5. UTI is often caused by ESBL-producing gram-negative bacteria such as *Escherichia coli (E. coli)*, *Klebsiella pneumoniae (K. pneumonia*) and *Pseudomonas aeruginosa (P. aeruginosa)* 6. Almost all these pathogens are Multi-Drug Resistant (MDR) and result in clinical problems because of limited therapeutic options 7,8. Extended-spectrum ß-lactamase antibiotics such as cefotaxime, ceftazidime and ceftriaxone are mostly used to treat this type of infection in most countries 9. Recently, the prevalence of antibiotic resistance in gram-negative bacteria which result in UTI has increased strongly 10,11. *Enterobacteriaceae* and some other gram-negative pathogens became highly ESBL-producing bacteria. ESßL encoding genes are located on bacterial DNA, plasmids or transposons that can transfer easily between two or more bacteria such as *E. coli*, *K. pneumoniae*, *Enterobactercloacae* (*E. cloacae)* and others 12,13.

Since the 1980s, ESβL antibiotics have been widely used for treatment of different infections caused by gram-negative bacteria such as burns infections, wound infections and UTI 14,15. However, bacterial resistance has originated fast due to the production of special enzymes called ESBLs 16. These enzymes are derived from some genes such as *Bla-TEM*, *Bla-SHV* and *Bla-CTX-M* for the narrower spectrum ß-lactamases by mutations that alter the amino acid configuration around the enzyme active site 17. They are typically encoded by plasmids that can be exchanged between bacterial species 18. It is also reported that the predominant types of ESBLs in *K. pneumoniae*, *P. aeruginosa* and *E. coli* and some others type of gram-negative bacteria isolated from urine of patients with UTI are *TEM* type followed by *SHV* and *CTX-M*. Usually, infections caused by ESBL-producing bacteria are associated with increased morbidity and mortality which entails enhanced healthcare costs 19,20.

In Iraq, there are no studies focusing on the relationship between the prevalence of ESLB-genes in gram-negative bacteria causing UTI isolation from urine of outpatients infected with CKD and Without Kidney Disease (WKD). Therefore, the main aim of the present study was to investigate the antibiotic susceptibility patterns and the distribution of *Bla-TEM*, *Bla-SHV* and *Bla-CTX-M* genes in gram-negative bacteria isolated from outpatients infected with UTI, with and without CKD in Al-Najaf city, Iraq.

## Materials and Methods

### Patients and study design

This was a case control study carried out in laboratory of University of Kufa, Faculty of Science, Department of Microbiology, Iraq. A total of 120 outpatients (males and females in different age groups) were included in this study who were infected with UTI and were divided in two groups, group one (control) including 60 outpatients infected with UTI WKD and group two (case study) including 60 patients infected with UTI with CKD (diagnosed by specialist physician according to abnormal urinary parameters and an increase in serum creatinine) during the period between July to December 2017.

### Urine samples collection

Ten *ml* of clean and mid-stream of urine samples were collected in sterile containers (Himedia-India) from two groups of outpatients who visited private clinics in Al-Najaf city. All containers were labeled according to gender and age of each patient. Immediately, the urine samples were processed for bacterial cultivation and identification 21.

### Isolation and identification of gram-negative bacterial isolates

All urine samples were inoculated by sterile loop (Himedia-India) immediately on blood agar plate and MacConkey agar plate (Oxoid, UK). All agar plates were incubated aerobically at 37*°C* for 24 *hr*. Colony Forming Units (CFUs) method was used for growing single and pure bacterial colony; all urine samples containing less than 10^5^*CFUs/ml* were excluded 22. All single and pure bacterial colonies were identified according to colony morphology, gram stain, lactose or non-lactose fermenter on MacConkey agar plate, capsule formation and according to standard biochemical tests such as motility, IMVIC, oxidase, catalase and TSI 21. In addition, all bacterial isolates were streaked on CHROM agar medium (Orientation, France). Final identification was done according to Vitek2® system (BioMerieux® -France).

### Antibiotic susceptibility test

Antibiotic susceptibility testing was done by Kirby-Bauer disc diffusion method 23. Five to three pure and fresh bacterial colonies were suspended in nutrient broth (Oxoid, UK) and adjusted to 0.5 Mc-Ferland standard tube (1.5×10^8^ *CFUs/ml*). By using sterile swab (Bioanalyse, Turkey), the suspension was streaked onto the surface of Mueller Hinton agar (Oxoid, UK). By sterile forceps, all antibiotics disc were placed onto the surface of Mueller Hinton agar (Oxoid, UK) and incubated aerobically at 37*°C* for 24 *hr*. Clinical and Laboratory Standards Institute 24 was used as a guideline of antibiotic susceptibility and resistance according to bacterial growth zone diameter. Twelve antibiotics used in the current study were obtained from Bioanalyse, Turkey; Amoxicillin 25 *μg* (AX), Amoxicillin+Clavulanic acid 30 *μg* (AMC), Cefotaxime 30 *μg* (CTX), Ceftriaxone 30 *μg* (CRO), Ceftazidime 30 *μg* (CAZ), Imipenem 10 *μg* (IMP), Gentamicin 15 *μg* (CN), Amikacin 30 *μg* (AK), Tobramycin 10 *μg*, Tetracycline 30 *UI* (TE), Ciprofloxacin 5 *μg* (CIP) and Levofloxacin 30 *μg* (LIV). *E. coli* ATCC-25922 was used as a control isolate. MDR, Extensive-Drug Resistance (XDR) and Pan-Drug Resistance (PDR) bacterial isolates were determined as follows; each bacterial isolate was resistant to three deferent antibiotics class considered as MDR, each remained susceptible to one or two antibiotics classes considered as XDR and some resistant to all antibiotics classes considered as PDR 25.

### Primary phenotypic detection of ESBL-producing bacterial isolates

This method was done according to Clinical and Laboratory Standards Institute (CLSI, 2006) 25. Standard antimicrobial susceptibility test was performed for each bacterial isolate of three antibiotics; ceftriaxone 30 *μg*, cefotaxime 30 *μg* and ceftazidime 30 *μg*, and any bacterial isolate showing zone of inhibition of *≤*25 *mm*, *≤*27 *mm* and *≤*22 *mm*, respectively was considered as ESBL-producing isolate.

### Confirmatory phenotypic detection of ESBL-producing bacteria

Double disc synergy test was done according to Sarojamma and Ramakrishna 26 and Aljanaby and Alhasnawi 2. Bacterial suspension was adjusted according to 0.5 Mc-Ferland standard tube (1.5×10^8^ *CFUs/ml*). Amoxicillin 10 *μg*+Clavulanic acid 20 *μg* was placed in the center of Mueller Hinton agar plate, around three sides of ceftriaxone 30 *μg*. Cefotaxime 30 *μg* and ceftazidime 30 *μg* were placed with distance of 15 *mm* to center of Amoxicillin 10 *μg*+Clavulanic acid 20 *μg* and the plates were incubated at 37*°C* for 24 *hr*. Any inhibition zone of any type of 3^rd^ generation cephalosporins antibiotics was increased towards Amoxicillin 10 *μg*+Clavulanic acid 20 *μg* which was considered as the positive result of the study.

### DNA extraction

This method was preformed according to Aljanaby and Alhasnawi 2 and Aljanaby and Medhat 7. Briefly, five fresh and pure bacterial colonies were suspended in 500 *μl* of distilled water and heated in water bath (Oxoid, UK) for 30 *min* and the supernatant was taken as a DNA template after centrifugation at 7500 *rpm* for 20 *min*.

### PCR primers and thermo cycling conditions

Three primer sequences for three genes and thermo cycle conditions were used in the current study as shown in tables 1 and 2, respectively. Five *μl* aliquots of PCR product were analyzed by gel electrophoresis with 2% agarose. Gel was stained with ethidium bromide at 2 *mg/ml* and visualized with UV light.

**Table 1.** Primers sequences of three genes used in this study

**Name of gene**	**Oligo sequence (3′→5′)**	**ProductSize (*bp*)**	**Reference**
***blaTEM***	F:CAGCGGTAAGATCCTTGAGA	643	
	R:ACTCCCCGTCGTGTAGATAA		
***blaSHV***	F:GGCCGCGTAGGCATGATAGA	714	Ensor *et al*, 27
	R:CCCGGCGATTTGCTGATTTC		
***blaCTX-M***	F:AACCGTCACGCTGTTGTTAG	766	
	R:TTGAGGCGTGGTGAAGTAAG		

**Table 2.** Thermo cycle conditions of three genes used in this study

**Gene**	**Initial denaturation *°C* / Time**	**Cycling condition *°C* / Time**				**Final extension *°C* /Time**	**Reference**

		**Denaturation**	**Annealing**	**Extension**	**Number of cycles**		
***TEM***	95 *°C* /5 *min*	94*°C* /30 *s*	52*°C*/45 *s*	72*°C*/45 *s*	30	72*°C* /7 *min*	Ensor *et al*, 27
***SHV***	95 *°C* /5 *min*	94*°C* /30 *s*	55*°C*/60 *s*	72*°C*/45 *s*	30	72*°C* /7 *min*	
**CTX-M**	95 *°C* /5 *min*	94*°C* /30 *s*	57*°C* /45 *s*	72*°C*/45 *s*	30	72*°C* /7 *min*	

### Statistical analysis

Chi-squared test was used for the comparison between samples using graph-pad prism computer software version 8. A p-value less than 0.05 was considered statistically significant.

## Results

### Patients

**Figure 1.**
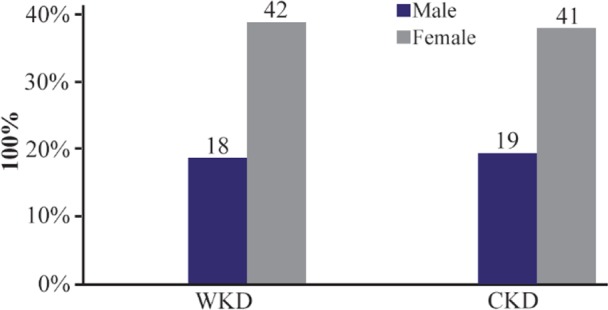
Distribution of 120 outpatients infected with urinary tract infection according to gender. WKD: Without kidney disease, CKD: Chronic kidney disease.

Out of 120 outpatients with UTI, the results indicated that there were 37 males; 18 (15%) WKD, 19 (15.8%) CKD and 83 females; 42 (35%) WKD, 41 (34.1%) with CKD (Figure 1). According to age groups, the results proved that the age group 51–60 years old was the most prevalent range among patients with UTI (48 patients 40%; 22 patients were WKD and 26 patients with CKD) (Table 3).

**Table 3.** Distribution of 120 outpatients infected with urinary tract infection according to age groups

**Ages (years)**	**WKD (100%)**	**CKD (100%)**	**Total (100%)**
**10–20**	3 (5)	4 (6.6)	7 (5.8)
**21–30**	7 (11.7)	5 (8.3)	12 (10)
**31–40**	10 (16.7)	7 (11.7)	17 (14.2)
**41–50**	14 (23.3)	16 (26.7)	30 (25)
**51–60**	22 (36.6)	26 (43.3)	48 (40)
**61–70**	4 (6.7)	2 (3.3)	6 (5)
**Total**	60 (100)	60 (100)	120 (100)

WKD: Without kidney disease, CKD: Chronic kidney disease.

### Total bacterial isolates

**Figure 2.**
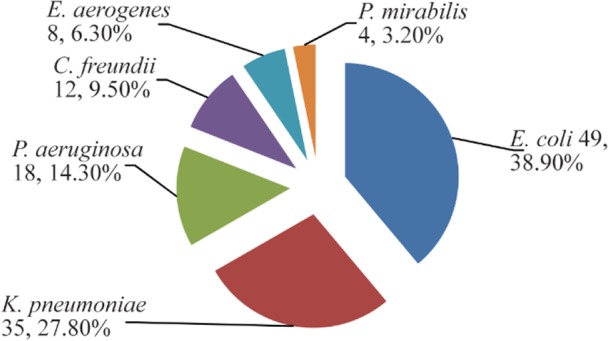
Numbers and percentages of gram-negative bacteria isolated from 120 urine samples of outpatients infected with urinary tract infection.

**Figure 3.**
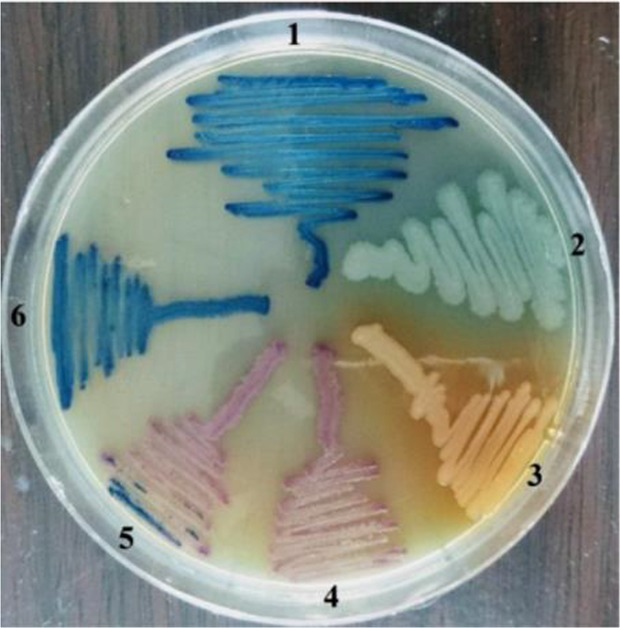
Gram-negative bacteria on CHROMagar surface after 24 *hr* of incubation at 37*°C* isolated from urine of outpatients infected with urinary tract infection. 1- *K. pneumoniae* 2- *P. aeruginosa* 3- *Proteus* 4- *E. coli* 5- *C. freundii* 6- *E. aerogenes*.

A total of 126 different gram-negative bacterial isolates were isolated from 120 urine samples of patients infected with UTI. *E. coli* was the most prevalent bacteria (49 isolates, 38.9%) followed by *K. pneumoniae* (35 isolates, 27.8%), *P. aeruginosa* (18 isolates, 14.3%), *Citrobacter freundii (C. freundii)* (12 isolates, 12.9%), *Enterobacter aerogenes (E. aerogenes*) (8 isolates, 6.3%) and *Proteus mirabilis (P. mirabilis)* (4 isolates, 3.2%) (Figures 2 and 3). The results demonstrated that there were 60 bacterial isolates from WKD patients and 66 isolates from CKD patients (Table 4).

**Table 4.** Numbers and percentages of gram-negative bacterial isolates from 120 urine samples of outpatients infected with urinary tract infection according to gender

**Bacteria**	**WKD**		**Total (100%)**	**CKD**		**Total (100%)**	**Total isolates (100%)**
	
	**Male**	**Female**		**Male**	**Female**		
***E. coli***	9	20	29 (48.3)	7	10+3 ^*^	20 (30.3)	49(38.9)
***K. pneumoniae***	6	12	18 (30)	5	9+3 ^*^	17 (25.8)	35(27.8)
***P. aeruginosa***	3	5	8 (13.3)	3	7	10 (15.2)	18(14.3)
***C. freundii***	0	3	3 (5)	3	6	9 (13.7)	12(9.5)
***E. aerogenes***	0	2	2 (3.4)	1	5	6 (9)	8(6.3)
***P.*** ***mirabilis***	0	0	0 (0.0)	0	4	4 (6)	4(3.2)
**Total**	18	42	60 (100)	19	47	66 (100)	126(100)

WKD: Without kidney disease, CKD: Chronic kidney disease,

* = Mixed growth

### Antibiotics susceptibility testing

**Figure 4.**
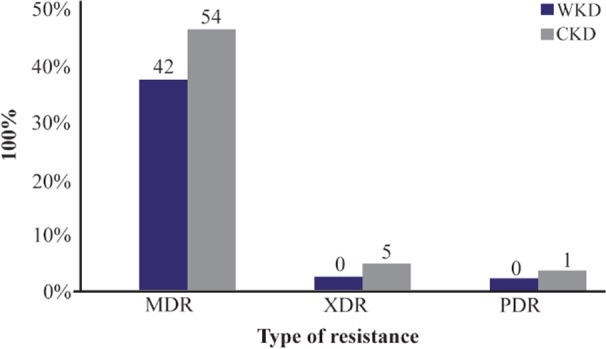
MDR, XDR and PDR gram-negative bacteria isolated from outpatients infected with urinary tract infection. MDR: Multidrug resistance; XDR: Extensive drug resistance; PDR: Pandrug resistance, WKD: Without kidney disease, CKD: Chronic kidney disease.

**Figure 5.**
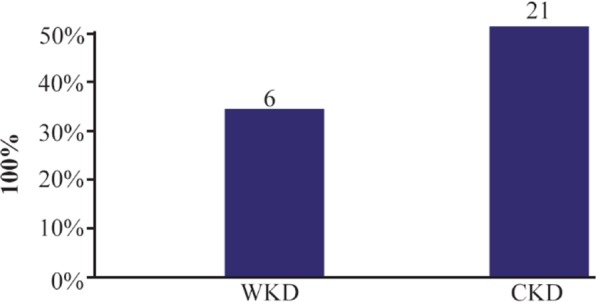
Prevalence of extended spectrum beta-lactamase-producing gram-negative bacteria isolated from outpatients infected with urinary tract infection. WKD: Without kidney disease, CKD: Chronic kidney disease.

**Figure 6.**
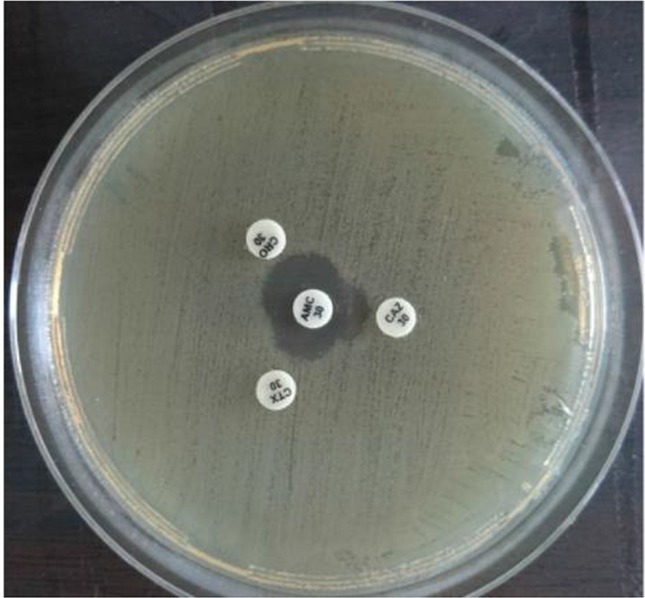
Positive result of ESBL-producing *K. pneumoniae* by DDST on Muller Hinton agar surface after 24 *hr* of incubation at 37*°C*. CRO: Ceftriaxone 30 *μg*.*,* CTX: Cefotaxime 30 *μg*, CAZ: Ceftazidime 30 *μg* AMC: Amoxi/Clavulanic acid 30 *μg*.

According to the results of antibiotics susceptibility test in tables 5 and 6, most bacterial isolates were highly resistant against most antibiotics especially against amoxicillin and third generation cephalosporins, while imipenem provided the best antibacterial effect against most isolates. Most bacterial isolates from urine of CKD patients were highly resistant to antibiotics as compared with isolates from urine of WKD patients. On the other hand, the results proved that there was a high incidence of MDR bacteria isolated from urine of CKD patients (54 isolates, 42.9%) as compared with WKD patients (42 isolates, 33.3%) (Figure 4) (Table 7). All CKD isolates were MDR with percentages between 80 to 95% except *E. aerogenes* and *P. mirabilis* which were 50%. *E. coli*, *K. pneumoniae* and *P. aeruginosa* isolated from urine of CKD patients were XDR with percentages of 5, 11.7 and 20%, respectively, and there was one *K. pneumoniae* isolate with 5.8% which was PDR (Table 7). The results showed that almost CKD isolates were ESBL-producing bacteria (21 isolates, 16.6%) as compared with WKD isolate (6 isolates, 4.7%) (Figure 5) and there was significant increase in CKD and WKD isolates in *E. coli* and *K.pneumoniae* with p-value= 0.0497 and 0.0153, respectively (Figure 6, Table 8).

**Table 5.** Antimicrobials sensitivity test of 126 gram-negative bacterial isolates from urine of outpatients infected with urinary tract infection

**AB**	***E. coli* 49 (100%)**			***K. pneumoniae* 35 (100%)**			***P. aeruginosa* 18 (100%)**		

	**S(100%)**	**I(100%)**	**R(100%)**	**S(100%)**	**I(100%)**	**R(100%)**	**S(100%)**	**I(100%)**	**R(100%)**
**AX**	10(20.4)	0(0.0)	39(79.6)	5(14.2)	0(0.0)	30(85.8)	0(0.0)	0(0.0)	18(100)
**AMC**	23(46.9)	2(4)	24(49.1)	9(25.8)	0(0.0)	26(74.2)	8(44.5)	0(0.0)	10(55.5)
**CTX**	28(57.2)	0(0.0)	21(42.8)	15(42.8)	0(0.0)	20(57.2)	10(55.5)	0(0.0)	8(44.5)
**CRO**	27(55.1)	0(0.0)	22(44.9)	12(34.3)	0(0.0)	23(65.7)	10(55.5)	0(0.0)	8(44.5)
**CAZ**	24(48.9)	3(6.2)	22 (44.9)	14(40)	0(0.0)	21(60)	9(50)	0(0.0)	9(50)
**IMP**	49(100)	0(0.0)	0(0.0)	33(94.3)	0(0.0)	2(5.7)	18(100)	0(0.0)	0(0.0)
**CN**	31(63.3)	0(0.0)	18(36.7)	14(40)	2(5.7)	19(54.3)	11(61.3)	0(0.0)	7(38.7)
**AK**	35(71.5)	0(0.0)	14(28.5)	23(65.7)	3(8.5)	9(25.8)	13(72.3)	0(0.0)	5(27.7)
**TM**	34(69.4)	0(0.0)	15(30.6)	23(65.7)	0(0.0)	12(34.3)	9(50)	0(0.0)	9(50)
**TE**	30(61.3)	0(0.0)	19(38.7)	17(48.5)	0(0.0)	18(51.5)	10(55.5)	0(0.0)	8(44.5)
**CIP**	34(69.3)	0(0.0)	15(30.6)	19(54.3)	0(0.0)	16(45.7)	11(61.3)	0(0.0)	7(38.7)
**LIV**	31(63.3)	0(0.0)	18(36.7)	21(60)	0(0.0)	14(40)	11(61.3)	0(0.0)	7(38.7)

**AB**	***C. freundii* 12 (100%)**			***E. aerogenes* 8 (100%)**			***P. mirabilis* 4 (100%)**		

	**S(100%)**	**I(100%)**	**R(100%)**	**S(100%)**	**I(100%)**	**R(100%)**	**S(100%)**	**I(100%)**	**R(100%)**
**AX**	2(16.6)	0(0.0)	10(83.4)	3(37.5)	0(0.0)	5(62.5)	1(25)	0(0.0)	3(75)
**AMC**	3(25)	1(8.6)	8(66.4)	5(62.5)	0(0.0)	3(37.5)	3(75)	0(0.0)	1(25)
**CTX**	6(50)	2(16.6)	4(33.4)	6(75)	0(0.0)	2(25)	2(50)	0(0.0)	2(50)
**CRO**	9(75)	0(0.0)	3(25)	6(75)	0(0.0)	2(25)	2(50)	0(0.0)	2(50)
**CAZ**	9(75)	0(0.0)	3(25)	5(62.5)	0(0.0)	3(37.5)	2(50)	0(0.0)	2(50)
**IMP**	12(100)	0(0.0)	0(0.0)	8(100)	0(0.0)	0(0.0)	4(100)	0(0.0)	0(0.0)
**CN**	9(33.3)	0(0.0)	8(66.4)	5(62.5)	0(0.0)	3(37.5)	3(75)	0(0.0)	1(25)
**AK**	9(75)	1(8.4)	2(16.6)	8(100)	0(0.0)	0(0.0)	4(100)	0(0.0)	0(0.0)
**TM**	9(75)	1(8.4)	2(16.6)	6(75)	0(0.0)	2(25)	3(75)	0(0.0)	1(25)
**TE**	8(66.6)	0(0.0)	4(33.4)	5(62.5)	0(0.0)	2(25)	3(75)	0(0.0)	1(25)
**CIP**	8(66.6)	0(0.0)	4(33.4)	5(62.5)	0(0.0)	3(37.5)	3(75)	0(0.0)	1(25)
**LIV**	8(66.6)	0(0.0)	4(33.4)	5(62.5)	0(0.0)	3(37.5)	3(75)	0(0.0)	2(50)

AB: Antibiotics, S: Sensitive, I: Intermediate, R: Resistance, AX: Amoxicillin 25 *μg* , AMC: Amoxicillin + Clavulanic acid 20+10 *μg* , CTX: Cefotaxime 30 *μg* , CRO: Ceftriaxone 30 *μg* , CAZ: Ceftazidime 30 *μg* , IMP: Imipenem 10 *μg* , CN: Gentamicin 15 *μg* , AK: Amikacin 30 *μg* , TM: Tobramycin 10 *μg* , TE: Tetracycline 30 *UI* , CIP: Ciprofloxacin 5 *μg* , LIV: Levofloxacin.

**Table 6.** Numbers and percentages of gram-negative bacteria that were resistant to antimicrobials isolated from urine of outpatients infected with urinary tract infection

**Antibiotics**	***E. coli***		***K. pneumoniae***		***P. aeruginosa***	

	**WKD No.(100%)**	**CKD No.(100%)**	**WKD No.(100%)**	**CKD No.(100%)**	**WKD No.(100%)**	**CKD No.(100%)**
**AX**	14(35.9)	25(64.1)	10(33.3)	20(66.7)	7(38.9)	11(61.1)
**AMC**	9(37.5)	15(62.5)	11(42.3)	15(57.7)	6(60)	4(40)
**CTX**	8(38)	13(62)	9(45)	11(55)	5(62.5)	3(37.5)
**CRO**	12(54.5)	10(45.5)	10(43.4)	13(56.6)	6(75)	2(25)
**CAZ**	8(36.3)	14(63.7)	10(47.6)	11(52.4)	4(44.4)	5(55.6)
**IMP**	0(0.0)	0(0.0)	00(0.0)	2(100)	00(0.0)	00(0.0)
**CN**	7(38.8)	11(61.2)	10(52.6)	9(47.4)	3(42.8)	4(57.2)
**AK**	8(57.1)	6(42.9)	4(44.4)	5(55.6)	2(40)	3(60)
**TM**	5(33.3)	10(66.7)	4(33.3)	8(66.7)	5(55.5)	4(44.5)
**TE**	7(36.8)	12(63.2)	11(61.1)	7(38.9)	2(25)	6(75)
**CIP**	6(40)	9(60)	6(37.5)	10(62.5)	2(28.5)	5(71.5)
**LIV**	8(44.4)	10(55.6)	9(64.2)	5(35.8)	3(42.8)	4(57.2)

**Antibiotics**	***C. freundii***		***E. aerogenes***		***P. mirabilis***	

	**WKD No.(100%)**	**CKD No.(100%)**	**WKD No.(100%)**	**CKD No.(100%)**	**WKD No.(100%)**	**CKD No.(100%)**
**AX**	3(30)	7(70)	1(20)	4(80)	0(0.0)	3(100)
**AMC**	3(37.5)	5(62.5)	1(33.3)	2(66.6)	0(0.0)	1(100)
**CTX**	2(50)	2(50)	0(0.0)	2(100)	0(0.0)	2(100)
**CRO**	0(0.0)	3(100)	1(50)	1(50)	0(0.0)	2(100)
**CAZ**	0 (0.0)	3(100)	2(66.6)	1(33.3)	0(0.0)	2(100)
**IMP**	0(0.0)	0(0.0)	0(0.0)	0(0.0)	0(0.0)	0(0.0)
**CN**	3(37.5)	5(62.5)	0(0.0)	3(100)	0(0.0)	1(100)
**AK**	0(0.0)	2(100)	0(0.0)	0(0.0)	0(0.0)	0(100)
**TM**	0(0.0)	2(100)	1(50)	1(50)	0(0.0)	1(100)
**TE**	1(25)	3(75)	1(50)	1(50)	0(0.0)	1(100)
**CIP**	1(25)	3(75)	1(33.3)	2(66.6)	0(0.0)	1(100)
**LIV**	2(50)	2(50)	0(0.0)	3(100)	0(0.0)	2(100)

WKD: Without kidney disease, CKD: Chronic kidney disease, AX: Amoxicillin 25 *μg* , AMC: Amoxicillin + Clavulanic acid 20+10 *μg* , CTX: Cefotaxime 30 *μg* , CRO: Ceftriaxone 30 *μg* , CAZ: Ceftazidime 30 *μg* , IMP: Imipenem 10 *μg* , CN: Gentamicin 15 *μg* , AK: Amikacin 30 *μg* , TM: Tobramycin 10 *μg* , TE: Tetracycline 30 *UI* , CIP: Ciprofloxacin 5 *μg* , LIV: Levofloxacin.

**Table 7.** Numbers and percentages of MDR, XDR and PDR gram-negative bacteria isolated from urine of outpatients infected with urinary tract infection

***E. coli***			
**Resistance**	**WKD 29(100%)**	**CKD 20(100%)**	**Total 49(100%)**
MDR	15 (51.7)	19 (95)	34(69.3)
XDR	0(0.0)	1(5)	1(2)
PDR	0(0.0)	0(0.0)	0(0.0)
***K. pneumoniae***			
**Resistance**	**WKD 18(100%)**	**CKD 17(100%)**	**Total 35(100%)**
MDR	18(100)	14(82.3)	32(91.4)
XDR	0(0.0)	2(11.7)	2(5.7)
PDR	0(0.0)	1(5.8)	1(2.8)
***P. aeruginosa***			
**Resistance**	**WKD 8(100%)**	**CKD 10(100%)**	**Total 18(100%)**
MDR	8(100)	8(80)	16(88.8)
XDR	0(0.0)	2(20)	2(11.1)
PDR	0(0.0)	0(0.0)	0(0.0)
***C. freundii***			
**Resistance**	**WKD 3(100%)**	**CKD 9(100%)**	**Total 12(100%)**
MDR	1(33.3)	8(88.8)	9(75)
XDR	0(0.0)	0(0.0)	0(0.0)
PDR	0(0.0)	0(0.0)	0(0.0)
***E. aerogenes***			
**Resistance**	**WKD 2(100%)**	**CKD 6(100%)**	**Total 8(100%)**
MDR	0(0.0)	3(50)	3(37.5)
XDR	0(0.0)	0(0.0)	0(0.0)
PDR	0(0.0)	0(0.0)	0(0.0)
***P. mirabilis***			
**Resistance**	**WKD 0(100%)**	**CKD 4(100%)**	**Total 4(100%)**
MDR	0(0.0)	2(50)	2(50)
XDR	0(0.0)	0(0.0)	0(0.0)
PDR	0(0.0)	0(0.0)	0(0.0)

MDR: Multidrug resistance; XDR: Extensive drug resistance; PDR: Pandrug resistance. WKD: Without kidney disease, CKD: Chronic kidney disease.

**Table 8.** Numbers and percentages of phenotypic results of extended-spectrum β-lactamase -producing gram-negative bacteria isolated from outpatients infected with urinary tract infection

***E. coli***				
	**WKD 29(100%)**	**CKD 20(100%)**	**Total 49(100%)**	p-value ^*^
ESBL	2(6.9)	6(30)	9(18.3)	0.0497^*^
Non-ESBL	27(93.1)	14(70)	40(81.7)	
Total	29(100)	20(100)	49(100)	
***K. pneumoniae***				
	**WKD 18(100%)**	**CKD 17(100%)**	**Total 35(100%)**	**p-value**
ESBL	3(16.6)	10(58.8)	13(37.1)	0.0153^*^
Non-ESBL	15(83.4)	7(41.2)	22(62.9)	
Total	18(100)	17(100)	35(100)	
***P. aeruginosa***				
	**WKD 8(100%)**	**CKD 10(100%)**	**Total 18(100%)**	**p-value**
ESBL	1(12.5)	3(30)	4(22.2)	0.5882
Non-ESBL	7(87.5)	7(70)	14(77.3)	
Total	8(100)	10(100)	18(100)	
***C. freundii***				
	**WKD 3(100%)**	**CKD 9(100%)**	**Total 12(100%)**	**p-value**
ESBL	0(0.0)	1(11.1)	1(8.3)	1.0000
Non-ESBL	3(100)	8(88.9)	11(91.7)	
Total	3(100)	9(100)	12(100)	
***E. aerogenes***				
	**WKD 2(100%)**	**CKD 6(100%)**	**Total 8(100%)**	**p-value**
ESBL	0(0.0)	1(16.6)	1(12.5)	1.0000
Non-ESBL	2(100)	5(83.4)	7(87.5)	
Total	2(100)	6(100)	8(100)	
***P. mirabilis***				
	**WKD 0(100%)**	**CKD 4(100%)**	**Total 4(100%)**	**p-value**
ESBL	0(0.0)	0(0.0)	0(0.0)	-
Non-ESBL	0(0.0)	4(100)	4(100)	
Total	0(0.0)	4(100)	4(100)	

ESBL: Extended-spectrum β-lactamase -producing bacteria, WKD: Without kidney disease, CKD: Chronic kidney disease, P-value

* : Comparison between WKD and CKD.

### Genotypic detection

**Figure 7.**
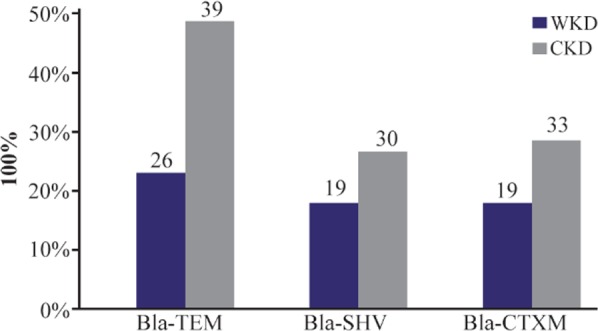
Distribution of ESBLs-genes of 126 gram-negative bacteria isolated from outpatients infected with urinary tract infections. WKD: Without kidney disease, CKD: Chronic kidney disease.

**Figure 8.**
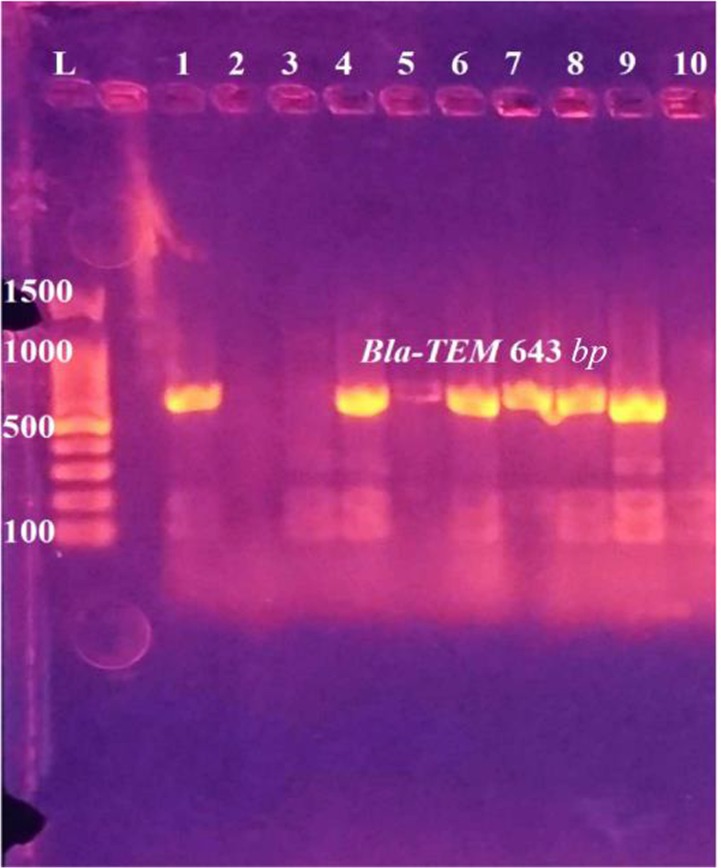
PCR amplification of *Bla-TEM* gene in *E. coli* isolates from urine of outpatients infected with UTI with chronic kidney disease showing positive results at 643 *bp*. L: DNA size marker. 1–10: number of isolates.

**Figure 9.**
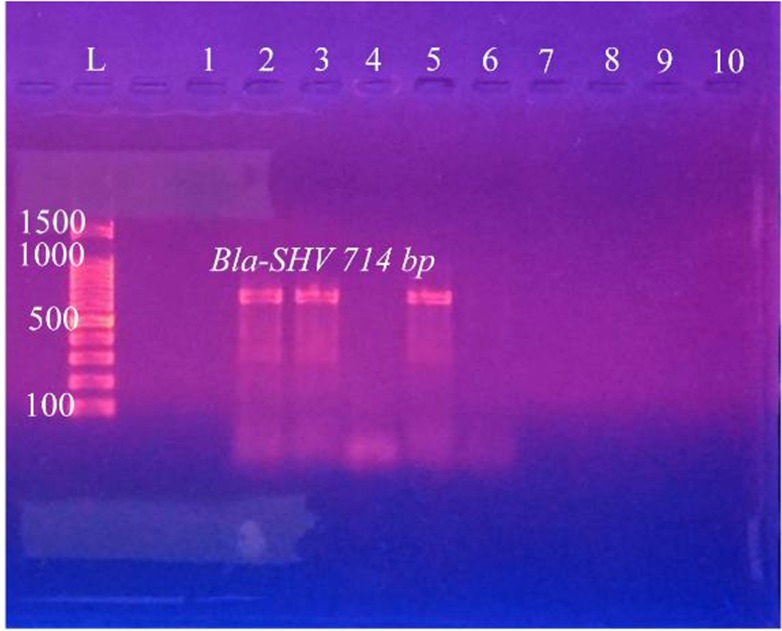
PCR amplification of *Bla-SHV* gene in *K. pneumoniae* isolates from urine of outpatients infected with UTI with chronic kidney disease showing positive results at 714 *bp*. L: DNA size marker. 1–10: number of isolates.

**Figure 10.**
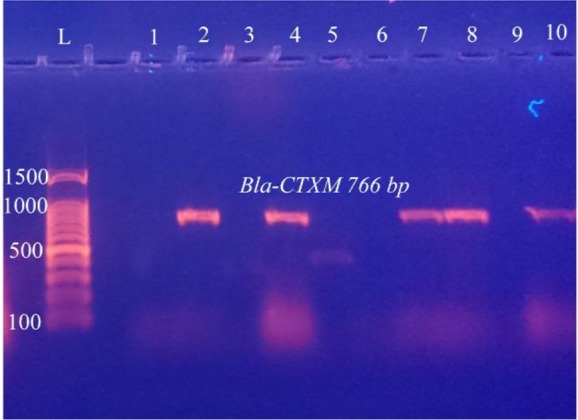
PCR amplification of *Bla-CTXM* gene in *C. freundii* isolates from urine of outpatients infected with UTI with chronic kidney disease showing positive results at 766 *bp*. L: DNA size marker. 1–10: number of isolates

Out of 126 total isolates, the results proved that all CKD isolates harbored ESBLs-genes more than WKD isolates. Thirty nine CKD isolates (46%) harbored *Bla-TEM*, 30 isolates (24%) harbored *Bla-SHV* and 33 isolates (26%) harbored *Bla-CTX-M* as compared with WKD isolates; 26 isolates (20.6%) harbored *Bla-TEM*, 19 isolates (15%) harbored *Bla-SHV* and *Bla-CTX-M* (Figure 7). *K. pneumoniae* was the one that mostly carried ESBLs-genes followed by *E. coli* and *C. freundii* (Table 9). PCR amplification of genes is shown in figures 8–10.

**Table 9.** Numbers and percentages of ESBLs-genes in 126 gram-negative bacterial isolates from outpatients infected with urinary tract infections

***E. coli***			
**Gene**	**WKD 29(100%)**	**CKD 20(100%)**	**Total 49(100%)**
*Bla-TEM*	9(31)	13(65)	21(42.8)
*Bla-SHV*	8(27.5)	10(50)	18(36.7)
*Bla-CTXM*	7(24.1)	12(60)	19(38.7)
***K. pneumoniae***			
**Gene**	**WKD 18(100%)**	**CKD 17(100%)**	**Total 35(100%)**
*Bla-TEM*	15(83.3)	15(88.2)	30(85.7)
*Bla-SHV*	10(55.5)	13(76.4)	23(65.7)
*Bla-CTXM*	11(61.1)	13(6.4)	24(68.5)
***P. aeruginosa***			
**Gene**	**WKD 8(100%)**	**CKD 10(100%)**	**Total 18(100%)**
*Bla-TEM*	0	1(10)	1(5.5)
*Bla-SHV*	0	1(10)	1(5.5)
*Bla-CTXM*	0	1(10)	1(5.5)
***C. freundii***			
**Gene**	**WKD 3(100%)**	**CKD 9(100%)**	**Total 12(100%)**
*Bla-TEM*	1(33.3)	5(55.5)	6(50)
*Bla-SHV*	1(33.3)	3(33.3)	4(33.3)
*Bla-CTXM*	1(33.3)	4(44.4)	5(41.6)
***E. aerogenes***			
**Gene**	**WKD 2(100%)**	**CKD 6(100%)**	**Total 8(100%)**
*Bla-TEM*	1(50)	3(50)	4(50)
*Bla-SHV*	0(0.0)	2(33.3)	2(25)
*Bla-CTXM*	0(0.0)	2(33.3)	2(25)
***P.* mirabilis**			
**Gene**	**WKD 0(100%)**	**CKD 4(100%)**	**Total 4(100%)**
*Bla-TEM*	0(0.0)	2(50)	2(50)
*Bla-SHV*	0(0.0)	1(25)	1(25)
*Bla-CTXM*	0(0.0)	1(25)	1(25)

WKD: Without kidney disease, CKD: Chronic kidney disease

## Discussion

UTI is one of the most important diseases that infect both males and females especially in age groups between 50 to 70 years old. In the current study, the results proved that out of 120 patients, there were 37 (30.8%) males and 83 (69.2%) females infected with UTI and 51–60 year old group was the most infected one among age groups with percentage of 40%. These results are in agreement with Zhang *et al’s* 28 and Hsiao *et al’s* 29 findings when they found a high prevalence of older patients (50 to 75 years old) infected with UTI. On the other hand, females are prone to infection with UTI more than males because of absence of prostatic fluid (Antibacterial activity) and the shortness of the urethra 30,31.

CKD is the continuous defect of renal functions 32. Diagnosis on the basis of abnormal urinary parameters and increase in serum creatinine are associated with increased risk of acute kidney disease and mortality 33. Urinary tract infection affects approximately up to 6 to 40% of CKD, being the main infectious complication among such patient population and one of the most common causes of CKD and mortality 34,35.

In this study, one of the most important results was a high incidence of MDR bacteria (54 isolates, 42.9%) and ESBL- producing gram-negative bacteria (21 isolates, 16.6%) among CKD patients as compared with WKD patients (42 isolates, 33.3% of MDR bacteria and 6 isolates, 26.1% of ESBL-producing gram-negative bacteria) among 126 different gram-negative bacterial isolates. *K. pneumoniae*, *E. coli* and *C. freundii* were the most virulent pathogens isolated from CKD patients. Also, all CKD isolates harbored *Bla-TEM*, *Bla-SHV* and *Bla-CTX-M* genes more than WKD isolates.

Recurrent infection with urinary tract was associated with septic shock or sepsis leading to renal failure or chronic kidney disease 36. On the other hand, some past studies proved that CKD was not a common complication in patients with renal failure 37,38. The risk of UTI in patients with CKD might be increased by host factors such as immunodeficiency and low urinary flow rate 39. Multi-drug resistance of uropathogenic gram-negative bacteria such as *K. pneumoniae*, *E. coli* and *C. freundii* is the leading cause in majority of UTI, including pyelonephritis which may lead to CKD and renal failure in healthy individuals 40. The development of UTI depends on anatomical barriers, host defense strategy and the virulence factors of uropathogenic bacteria that can stay within the urinary tract and act as a reservoir for recurrent UTI and many dangerous complications 41.

The main causative agents responsible for UTI are gram-negative bacteria mostly, *E. coli* and *k. pneumoniae* with percentage about 40–60% and 35–25%, respectively, followed by *C. freundii*, *E. aerogenes* and *P. mirabilis* 42–44. *K. pneumoniae* and virulence strains of *E. coli* are the most important uropathogenic bacteria causing UTI 2. They are among the most common MDR bacteria causing recurrent UTI 45. Recently, *K. pneumonia*, *E. coli*, *C. freundii* and most gram-negative bacteria have shown their ability to acquire plasmid encoding for *ESBL* genes such as *Bla-TEM*, *Bla-SHV* and *Bla-CTX-M* and become highly resistant to different antibiotics and wide-spectrum of 3^rd^ generation cephalosporins in hospitals and in community 5,13,46. About more than 200 different types of ESBLs have been discovered worldwide. Most of these genes are found in *Enterobacteriaceae* family especially in *K. penumoniae* 47. *TEM* and *SHV* are the ESBLs mostly found 48,49. Later, *CTX-M* was discovered in Germany in 1989 and mostly found in *K. pneumoniae*, *E. coli* and in other *Enterobacteriaceae* family 47,50. In the current study, almost all *P. aeruginosa* isolates from CKD patients were MDR with high resistance to antibiotics as compared with those isolates from WKD patients, but they harbored small numbers of *ESBL* genes. Livermore and Subedi *et al* suggested that *P. aeruginosa* carries multi-resistance plasmids less than *K. pneumoniae* and other members of *Enterobacteriaceae* family and develops resistance to cephalosporins due to mutational and intrinsic and acquired mechanisms 51,52.

Extended spectrum beta lactamases are enzymes not able to hydrolyze carbapenem and cephamycins but able to hydrolyze 3^rd^ and 4^th^ generation cephalosporins. These enzymes present a public health concern due to the high incidence in some members of *Enterobacteriaceae* family such as *E. coli* and *K. pneumoniae* in both hospitals and community 2,5,53. Some high virulence strains of gram-negative bacteria such as *E. coli*, *K. pneumoniae* and *C. freundii* are associated with UTI and if they remain untreated, these strains become capable of adhesion and colonization in the urinary tract of human and migrate to the bladder to cause cystitis and acute pyelonephritis and ultimately cause kidney damage and chronic kidney disease.

## Conclusion

This was the first study in Iraq focused on bacterial isolates from urine of UTI patients infected with chronic kidney disease. This study suggested that all bacterial isolates from those patients were highly resistant to antibiotics and were more virulent as compared with the same isolates from urine of UTI patients without kidney disease. The reason may be related to ignoring the treatment of UTI or overusing antibiotics. Therefore, it is advised to be more careful regarding recurrent infection of UTI because this recurrent infection may cause dangerous complications in kidney such as chronic kidney disease or renal failure.
